# F77. OXYTOCIN ENHANCES VISUAL ATTENTION TO FACIAL STIMULI IN PATIENTS WITH SCHIZOPHRENIA: EVIDENCE FROM AN EYE-TRACKING STUDY

**DOI:** 10.1093/schbul/sby017.608

**Published:** 2018-04-01

**Authors:** Lilla Porffy, Rebekah Wigton, Antoine Coutrot, Dan Joyce, Isabelle Mareschal, Sukhi Shergill

**Affiliations:** 1 Institute of Psychiatry, Psychology & Neuroscience; 2 Beth Israel Deaconess Medical Center; 3 University College London; 4 Queen Mary University of London

## Abstract

**Background:**

Deficits in social cognition often develop during the prodromal stages of psychosis, remain stable over the course of the illness, and have a dramatic impact on daily functioning (Fett et al., 2011). Social cue processing, particularly face perception, plays a critical role in social cognitive functioning. Patients with schizophrenia struggle to extract information from faces and interpret facial expressions (Kohler et al., 2010). These deficits may be explained by restricted visual attention. Indeed, eye-tracking studies have demonstrated that people with schizophrenia show reduced exploratory behaviour (i.e. reduced number of fixations and longer fixation durations) in response to facial stimuli compared to healthy controls (e.g. Manor et al., 1999). Oxytocin has been demonstrated to exert pro-social effects on behaviour and modulate eye gaze during perception of faces. In the present study, we tested whether the neuropeptide, oxytocin, has a compensatory effect on visual processing of human faces.

**Methods:**

Twenty right-handed male subjects with schizophrenia (n = 16) or schizoaffective disorder (n = 4) were administered intranasal oxytocin 40UI or placebo in a double-blind, placebo-controlled, cross-over fashion during two visits separated by 7 days. Participants engaged in a free-viewing eye-tracking task, during which they were looking at 6 facial images of two Caucasian men displaying angry, happy, and neutral facial expressions, and 6 control images in a random order. Eye-tracking measures including 1) total number of fixations, 2) dispersion, 3) saccade amplitude, and 4) mean duration of fixations were captured using the EyeLink 1000 system (SR Research Ltd, Ottawa, Ontario, Canada). Four separate 2 x 4 repeated-measures analysis of variance (ANOVA) were carried out to evaluate the within-subject effects of treatment, stimuli, and the interactions between stimuli and treatment (p < .05, two-tailed).

**Results:**

We found a main effect of treatment (F1,17 = 16.139, p = .001), but not a main effect of stimuli (F3,51 = 1.479, p > .231) on total number of fixations. There was a main effect of treatment on duration of fixation, (F1,13 = 5.455, p = .036) but not a main effect of stimuli (F3,39 = 1.267, p = .299). For dispersion, there was a significant main effect of stimuli (F3,51 = 3.424, p = .024) but no main effect of treatment (F1,17 = 3.170, p = .093). Analysis of saccade amplitudes revealed no main effect of treatment (F1,17 = 2.666, p = .121) or stimuli (F3,51 = 0.289, p = .833). None of the interactions reached significance.

**Discussion:**

To our knowledge, this is the first study to explore the effects of oxytocin on eye movements in individuals with schizophrenia. We found that oxytocin increased exploratory viewing behaviour in response to affective facial stimuli by significantly increasing the total number and duration of fixations compared to placebo. While previous findings regarding oxytocin have been inconsistent, our findings are in line with research showing that the intranasal administration of 40UI oxytocin may improve social cognitive deficits in schizophrenia (e.g. Davis et al., 2013). Future experiments may wish to explore the correlation between eye movement changes induced by oxytocin and facial affect recognition in larger samples.

